# Modeling the Interplay Between Perceived Teacher Confirmation, L2 Grit and Well-Being Among Chinese EFL Students

**DOI:** 10.3390/bs16081397

**Published:** 2026-08-14

**Authors:** Xiaojing Hu, Jingke Xu, Haizi Wang

**Affiliations:** 1 School of Education, Xi’an International Studies University, Xi’an 710128, China; 107242025200853@stu.xisu.edu.cn; 2 School of Chinese Studies and Central Asia, Xi’an International Studies University, Xi’an 710128, China; 107242025000515@stu.xisu.edu.cn

**Keywords:** well-being, teacher confirmation, L2 grit, EFL learners, language-learning psychology

## Abstract

Studies on language-learning psychology demonstrate that perceived teacher confirmation may contribute to student motivation, grit and engagement. However, little attention has been paid to investigating the role of perceived teacher confirmation in language learners’ well-being, including those who learn English as a foreign language. Moreover, the potential working mechanism between perceived teacher confirmation and student well-being remains unsolved. In order to address these gaps, the current study aimed to investigate to what extent teacher confirmation may contribute to EFL learners’ sense of well-being via the mediating role of L2 grit. A total of 440 Chinese college-level EFL learners were recruited through convenience sampling to complete an online composite questionnaire. Pearson correlation and structural equation modeling were employed for statistical analyses. The results were as follows: (1) teacher confirmation was positively associated with Chinese EFL learners’ well-being; (2) L2 grit mediated the relationship between perceived teacher confirmation and EFL learners’ well-being. The research findings reveal that the employment of teacher confirmation behaviors in EFL settings may help to develop learners’ perseverance and sustain their interest in learning a language and, in turn, enhance their sense of well-being. Drawing on the findings, pedagogical implications were systematically discussed.

## 1. Introduction

Well-being is the essential foundation for students’ development, a key indicator of the quality of education, and the ultimate goal of education itself (Mercer, 2021; Pan et al., 2023). Learners exhibiting greater satisfaction with school life tend to generate significantly more positive learning experiences, whereas those with lower levels of well-being face greater risks of dropout and behavioral difficulties (Arslan & Renshaw, 2018). Well-being is recognized as a crucial factor influencing students’ self-confidence, growth mindset, motivation, and engagement (e.g., Datu & King, 2018; Seligman et al., 2009; Suldo et al., 2011). Students who enjoy high levels of well-being outperform their peers academically and are more likely to flourish across every domain of life (Kirkcaldy et al., 2004; Klapp et al., 2024).

Against the backdrop of the “affective turn” in applied linguistics, well-being has become a core construct in foreign language education (Gao et al., 2025; Mercer, 2021). In language education contexts, well-being refers to students’ ability to maintain emotional and psychological balance while navigating the complexities of acquiring a new language (Oxford, 2016). Well-being not only facilitates the immediate language-learning process but also exerts lasting positive effects on learners’ life orientations, personality traits, and emotion regulation capacities, which indirectly result in successful learning (Ergün & Demirdag, 2023; Shafiee Rad & Hashemian, 2023). Hence, the aim of language instruction should extend beyond developing learners’ linguistic knowledge and skills to include learners’ mental health and well-being (MacIntyre et al., 2019).

Well-being operates as both a protective factor against stress and a facilitator of active learning (Derakhshan & Datu, 2025). Research has shown that well-being is significantly and positively associated with language learners’ favorable outcomes. Specifically, L2 learners who experience a sense of well-being exhibit a higher level of enjoyment (Ergün & Demirdag, 2023). They tend to show more behavioral, cognitive and emotional engagement in learning a foreign language (Derakhshan & Datu, 2025; Pan et al., 2023; Shi & Sun, 2025) and display greater willingness to participate in extracurricular, informal English-learning activities (Zadorozhnyy & Lee, 2024). Well-being was also found to be a predictor of foreign language achievement among Saudi EFL female students (Alqarni, 2022). Likewise, well-being exerts an indirect effect on L2 achievement by regulating learners’ motivation among learners of languages other than English (L. Li & Liu, 2023). That is, learners’ sense of well-being in the learning process serves to trigger, sustain, and intensify EFL learners’ motivation, thereby promoting subsequent language development. Therefore, it is quite necessary to explore the potential antecedents of EFL learners’ well-being.

Teacher confirmation, conceptualized as “the transactional process by which teachers talk and interact with students that make them feel they are valuable and significant individuals” (Ellis, 2000, p. 265), is crucial for effective teacher–student communication. Students who perceive higher levels of teacher confirmation tend to achieve more in learning (Ellis, 2000, 2004), make more effort (Campbell et al., 2009), and demonstrate a higher level of satisfaction (Goodboy & Myers, 2008). In L2 contexts, teacher confirmation may contribute to learners’ perseverance and passion (Bai & Zheng, 2024), academic engagement (Liu et al., 2024; Y. Wang & Kruk, 2024), willingness to attend the EFL class (Y. Wang & Derakhshan, 2023), empowerment (M. Li & Chu, 2023), and language learning (Derakhshan, 2022).

Although a growing body of research has addressed the role of teacher confirmation in various L2 learning outcomes, its connection to EFL learners’ well-being has received limited empirical attention. In particular, whether and how teacher confirmation shapes well-being in the language classroom remains largely unsolved. Bridging this gap, the present research offers a novel perspective by hypothesizing that teacher confirmation operates through L2 grit, defined as perseverance and passion for long-term language-learning goals (Teimouri et al., 2022). Our study, therefore, probes whether this relationship contributes to enhanced well-being, with L2 grit serving as a key explanatory mechanism. Such an investigation responds to the growing call in applied linguistics for deeper inquiry into the underlying processes that facilitate effective language development (Fathi et al., 2024).

The potential mediating role of L2 grit in the relationship between teacher confirmation and well-being is supported by the literature. Empirically, recent findings indicating the contribution of teacher confirmation to sustained effort and enthusiasm (e.g., Tang & Zhu, 2024; Tu & Shi, 2024; Yang, 2021) and the contribution of L2 grit to well-being in L2 education (e.g., Zhou, 2023) offer a basis for inferring the mediating function of L2 grit in the link between teacher confirmation and EFL well-being.

Theoretically, the potential interplay of teacher confirmation, L2 grit and well-being could be explained by the self-determination theory (Ryan & Deci, 2020) and the rhetorical/relational goal theory (Mottet et al., 2006). According to the self-determination theory, when people’s basic psychological needs, namely, autonomy, competence, and relatedness, are satisfied, these need-satisfactions function as inherent catalysts for personal growth, social integration, and psychological well-being. In school contexts, the fulfillment of learners’ basic psychological needs may vitalize learners’ intrinsic motivation, thereby bolstering their academic engagement (Niemiec & Ryan, 2009). Next to learners, teachers are considered the most influential agents in facilitating effective classroom teaching and learning. A confirming instructional climate, cultivated through positive teacher communication behaviors (i.e., teacher confirmation), enables students to feel recognized as significant and valued classroom participants (Burns et al., 2018) and satisfies their fundamental psychological needs (Liu et al., 2024), which, in turn, promotes their achievement, long-term development, and overall well-being (Ryan & Deci, 2020).

The rhetorical/relational goal theory also provides a theoretical foundation for the current study. According to this theory, students have rhetorical and relational needs and wants, and successful learning may take place when teachers employ interpersonal behaviors to satisfy learners’ academic and relational needs and wants. Teachers’ employment of proper communication behaviors could initiate and sustain the positive ties between teachers and students (Rudick & Golsan, 2014). When teachers use suitable interpersonal behaviors to satisfy students’ relational and rhetorical needs, a constellation of favorable educational outcomes emerges, such as enhanced learning, heightened interest, deeper engagement, greater empowerment, stronger motivation, and increased achievement (Houser & Hosek, 2018).

Based on empirical findings and the theoretical assumptions mentioned above, we hypothesize that teachers’ confirming behaviors may empower students to demonstrate unwavering commitment and sustained interest in language learning and, in turn, promote their sense of well-being. To date, research on the well-being of language learners remains in its early stages (Mercer & Murillo-Miranda, 2025). The present study addresses this gap by probing how teacher confirmation (an external factor) and L2 grit (an internal factor) function both independently and in concert to shape EFL learners’ well-being. In terms of pedagogical practice, our results offer valuable directions for developing evidence-based interventions aimed at enhancing well-being in L2 settings.

## 2. Literature Review

### 2.1. Well-Being

The concept of well-being has been mainly conceptualized from the hedonic and eudaimonic perspectives. The hedonic perspective places an emphasis on the experience of happiness, with subjective well-being serving as the key construct in this area (Diener et al., 1999). Specifically, it reflects the individual’s perceived balance between positive and negative emotions, as well as their overall sense of life satisfaction. The eudaimonic perspective on well-being is concerned with “living in accordance with one’s true self and realizing one’s unique potentials in a life affording purpose, meaning, and growth” (Ryff & Singer, 1998, p. 25).

The PERMA model proposed by Seligman (2011) is an integration of both hedonic and eudaimonic perspectives, which is also used in ELT studies (e.g., L. Li & Liu, 2023; MacIntyre et al., 2019). The PERMA model comprises five core dimensions: positive emotion, engagement, relationships, meaning, and achievement. Positive emotion pertains to hedonistic well-being and refers to feelings of joy, pleasure and hope. Engagement refers to a psychological state of flow, in which a person loses a sense of time whilst involved in tasks or interactions. Positive relationships represent the social support and networks that are the foundations of societal life. A sense of meaning pertains to eudaimonic well-being and refers to a sense of purpose or direction in life. A sense of accomplishment involves a sense of competency in reaching one’s goals or achieving mastery in tasks. This model portrays well-being as a socially regulated individual construct that simultaneously emphasizes positive emotional experiences and meaningful life events (Mercer, 2021).

Although the significance of well-being is widely acknowledged, how to cultivate and maintain student well-being in L2 settings remains an unresolved issue. To respond to it, a growing body of research has been conducted to investigate its antecedents. Yet, these inquiries have primarily focused on the role of personal resources in enhancing students’ well-being, such as grit, mindfulness, resilience, motivation, critical thinking, self-efficacy, self-regulation, emotions, emotion regulation, and a growth mindset (e.g., Fan & Cui, 2024; Gao et al., 2025; Ma et al., 2025; Pikhart & Klimova, 2020; Rezai et al., 2024; Sadeghi & Abolfazli Khonbi, 2018; Tu & Shi, 2024; X. Wang & Wu, 2025; Zarrinabadi et al., 2022). Moreover, sporadic research has examined the role of teacher-related factors, such as teacher support (e.g., Derakhshan & Fathi, 2024; Pan et al., 2023; Tang & Zhu, 2024), teaching styles (Amorati & Hajek, 2025), and teachers’ positive interpersonal behaviors (i.e., teacher care) (Zhou, 2023) in enhancing student well-being. However, to the best of our knowledge, no research has yet examined the role of teacher confirmation in L2 learners’ well-being, nor the underlying mechanisms through which this relationship functions.

### 2.2. Teacher Confirmation

The concept of confirmation stems from Buber (1965), who described confirmation as any behavior that causes another to feel recognized, acknowledged, or endorsed. Confirmation is widely studied in various contexts, such as parent–adolescent relationships and development, health communication among romantic partners, teacher–student relationships, and coach–athlete relationships (Dailey, 2023). Teacher confirmation was first introduced by Ellis (2000, 2004) to the instructional context, which is concerned with instructor-initiated communicative acts that validate students as significant contributors to the classroom experience. It is widely acknowledged that teacher confirmation consists of three major dimensions, namely, “responding to students’ questions and/or comments,” “demonstrating interest in the student’s learning process,” and “employing an interactive teaching style in the classroom” (Ellis, 2000, p. 266). The first dimension relates to how much time teachers devote to answering students’ questions effectively and to what extent teachers take time to listen to students’ questions carefully. The second dimension is concerned with teachers’ interest in students’ learning process, or whether teachers are sufficiently enthusiastic about their students’ study process. The third dimension refers to the extent to which teachers employ interactive teaching methods to assist students in understanding course content. Employing an interactive teaching style, teachers can modify their teaching practices based on their students’ needs and interests.

In general education, instructor-deployed confirming behaviors were found to be positively associated with students’ self-reported emotional outcomes and satisfaction. Students who perceive higher levels of teacher confirmation are more likely to be motivated and have higher levels of self-efficacy beliefs about their learning abilities (Goldman et al., 2018; Peaslee, 2018) and participate more actively in the class (e.g., Campbell et al., 2009; Goodboy & Myers, 2008; LaBelle & Johnson, 2020). In addition, they experience less academic apprehension and are willing to talk in the class (Ellis, 2004; Hsu & Huang, 2017; Goldman et al., 2018). Teacher confirmation has also been identified as a positive antecedent of students’ learning, academic achievement and effective instruction (e.g., Ellis, 2000; Goldman et al., 2018; Goodboy & Myers, 2008; Schrodt et al., 2006) as well as their mental health and well-being (e.g., LaBelle & Johnson, 2021).

The concept of teacher confirmation underscores the importance for educators, particularly in the EFL context, to take an active role in guiding students’ learning, acknowledging their efforts, and fostering a supportive and positive classroom environment (Liu et al., 2024). As a key relational behavior, teacher confirmation plays a crucial role in L2 learners’ empowerment (Derakhshan, 2022; M. Li & Chu, 2023). L2 learners who perceive higher levels of teacher confirmation are more likely to feel empowered in instructional-learning contexts. Students tend to be more perseverant and persistent when they encounter difficulties and setbacks in language learning (Bai & Zheng, 2024), are more willing to attend EFL classes (e.g., Y. Wang & Derakhshan, 2023), and show more engagement in language-learning practice (e.g., Y. Wang & Kruk, 2024). Perceived teacher confirmation is also identified as a predictor of students’ affective, cognitive, and behavioral learning (Derakhshan, 2022). In addition, perceived teacher confirmation contributes to the fulfillment of students’ three basic psychological needs, namely, competence, autonomy and relatedness (Liu et al., 2024), all of which are of great significance in predicting students’ academic achievement, life-long development and well-being (Ryan & Deci, 2020).

Despite growing interest in teacher confirmation in L2 contexts recently, little empirical attention has been paid to how teacher confirmation shapes L2 learners’ well-being, let alone the pathways through which this relationship may occur. The present study aims at filling this lacuna by investigating the role of teacher confirmation in EFL learners’ sense of well-being with L2 grit as a mediator. The rationale for including L2 grit as a mediator is presented in the next section.

### 2.3. L2 Grit as the Potential Mediator

L2 grit refers to an individual’s “perseverance and interest” in learning a second or a foreign language in order to achieve their long-term goals regardless of difficulties and setbacks (Teimouri et al., 2022). Learners endowed with grit typically manifest an enduring dedication to their educational pursuits and a capacity to persevere throughout the challenging odyssey of second-language acquisition (Botes et al., 2025). This can, in turn, enable them to effectively confront potential challenges, setbacks, and obstacles.

L2 grit encompasses two facets, namely, perseverance of effort (PE) and consistency of interests (CI) (Teimouri et al., 2022). PE deals with the learner’s willingness to expend demanding, prolonged effort, while CI highlights the capacity to keep one’s curiosity alive even when confronted with obstacles and setbacks in language learning. L2 grit plays a pivotal role in predicting various desirable educational outcomes. For instance, L2 grit has a positive effect on student engagement (Sadoughi & Hejazi, 2023), foreign language learning enjoyment (Khajavy & Aghaee, 2024; Pawlak et al., 2025), and willingness to communicate with the target language (Lee, 2022). Moreover, multiple studies, including two meta-analyses (Cheng & Cui, 2024; Demir, 2024), have identified L2 grit as an antecedent of better language achievement (Sudina & Plonsky, 2021; Teimouri et al., 2022; Teimouri, 2025). Research also indicates that L2 grit reduces foreign language learning burnout (X. Hu et al., 2025) and exerts positive effects on EFL learners’ overall well-being (Tang & Zhu, 2024; Tu & Shi, 2024; Yang, 2021).

Just as mentioned in the Introduction part, the self-determination theory and the rhetorical/relational goal theory legitimize the treatment of L2 grit as a mediator through which teacher confirmation shapes student well-being. Empirical studies also provide evidence of the mediating role of L2 grit in the interplay between teacher confirmation and students’ well-being. For instance, in a recent study, Bai and Zheng (2024) examined the role of teacher confirmation in L2 grit among 309 low-proficiency university-level Chinese EFL learners. The results revealed that teacher confirmation is a positive predictor of EFL learners’ L2 grit. In addition, a growing body of research highlights the positive effect of L2 grit on EFL learners’ well-being. For example, Tang and Zhu (2024) found that L2 grit contributed to psychological well-being among 968 Chinese EFL learners. This may be because L2 grit, rooted in passion and perseverance, bolsters resilience and adaptability in language learning, which ultimately promotes students’ well-being. In a similar vein, Yang (2021) as well as Tu and Shi (2024) probed the role of L2 grit in influencing Chinese EFL learners’ well-being. Both studies showed that L2 grit significantly contributes to EFL students’ well-being in the Chinese educational context.

All in all, despite these valuable contributions, how far teacher confirmation may shape EFL learners’ well-being remains unknown. Moreover, the potential working mechanism of L2 grit between teacher confirmation behaviors and EFL learners’ well-being has been unexplored. To address these issues, the current study aims to explore the interactions between perceived teacher confirmation, L2 grit and well-being. Aligned with these objectives, the following two research questions are proposed:

(1) Does students’ perceived teacher confirmation significantly predict well-being among Chinese university-level EFL learners?

(2) Does L2 grit mediate the association between students’ perceived teacher confirmation and well-being among Chinese university-level EFL learners?

## 3. Method

### 3.1. Participants

Through convenience sampling, a group of 440 students participated in the current study. They were recruited from four universities in three provinces in China. The sample consisted of approximately 15.5% male students (n = 68) and 84.5% female students (n = 372). The average age of participants was 19.67 years (SD = 1.973). In addition to gender and age, optional demographic data were collected, including length of English learning, academic year, majors and overseas English learning experience. Their mean length of English study was 11.04 years. In total, 53.2% (n = 234) of the participants were first-year undergraduates, 18.2% (n = 80) were second-year undergraduates, 8% (n = 35) were third-year undergraduates, 2.7% (n = 12) were fourth-year undergraduates, and 18% (n = 79) were graduates. The sample comprised 295 English majors (67%) and 145 non-English majors (33%). With the exception of four participants, the students had no prior experience studying overseas.

### 3.2. Instruments

A composite questionnaire was used in the current study to collect the data. The composite questionnaire consisted of two parts. The first part included participants’ demographic information, such as age, gender, academic year, length of English learning, major, and overseas English learning experience. The second part incorporated three psychometrically validated instruments, namely, the Teacher Confirmation Scale, L2 Grit Scale and Student Well-Being Scale. All questionnaire items were presented in Chinese, which was the mother tongue of the participants. The first author produced an initial Chinese translation of all three scales. Two bilingual English teachers then independently back-translated the Chinese version into English. All authors jointly reviewed and compared the original and back-translated versions and resolved any inconsistencies through team discussion until full agreement was achieved. The following is a detailed introduction to the scales.

### 3.3. Teacher Confirmation Scale (TCS)

The Teacher Confirmation Scale, developed and validated by Ellis (2000), was used to measure Chinese EFL learners’ perceptions of teacher confirmation. “My English teacher” was inserted at the beginning of each original item to contextualize it. The scale consisted of three dimensions, namely, (a) teachers’ response to students’ questions/comments, (b) demonstrated interest in students and in their learning, and (c) teaching style (Ellis, 2000, p. 286). There were 16 items, ranging from 1 (strongly disagree) to 5 (strongly agree). Some example items in the scale were as follows: “My English teacher takes time to answer students’ questions fully” (item 1), “My English teacher communicates that he/she is interested in whether students are learning” (item 6) and “My English teacher uses an interactive teaching style” (item 12). The reliability coefficient reported for this questionnaire by Ellis (2000) was as high as 0.95. In Goldman and Goodboy’s (2014) study, the questionnaire yielded a Cronbach’s alpha of 0.92. In the current study, the TCS demonstrated good construct validity (x^2^/df = 3.672; CFI = 0.968; TLI = 0.961; SRMR = 0.025; RMSEA = 0.078) as well as excellent reliability (Cronbach’s α = 0.975).

### 3.4. L2 Grit Scale (L2GS)

EFL learners’ L2 grit was measured by Teimouri et al. (2022). The scale had 9 items, including two dimensions, namely, PE (5 items) and CI (4 items). Participants’ levels of L2 grit were assessed on a 5-point Likert scale ranging from 1 (strongly disagree) to 5 (strongly agree). The following were some example items: “I am a diligent English language learner” (item 1), “When it comes to English, I am a hard-working learner” (item 3), “I will not allow anything to stop me from my progress in learning English” (item 6), and “I put much time and effort into improving my English language weaknesses” (item 9). This scale has been widely used and validated in L2 settings (Teimouri, 2025). In this study, the first item in CI (i.e., “I am not as interested in learning English as I used to be”) was removed due to its low factor loading (<0.30) (Hair et al., 2019). The reliability alpha reported for this scale by Teimouri (2025) was 0.86. In the current study, the L2GS demonstrated good construct validity (x^2^/df = 3.143; CFI = 0.981; TLI = 0.968; SRMR = 0.044; RMSEA = 0.070) as well as good reliability (Cronbach’s α = 0.820).

### 3.5. Student Well-Being Scale (SWS)

Chinese EFL learners’ well-being was measured with the PERMA Profiler developed and validated by Butler and Kern (2016). The PERMA Profiler is a 23-item survey with 15 main items and 8 other items that can be used as filler items or be analyzed separately. The present study used 15 main items, echoing the five elements included in the PERMA model: positive emotion, engagement, positive relationships, meaning, and accomplishment. Each element was measured with three items on a 5-point Likert scale ranging from 1 (strongly disagree) to 5 (strongly agree). Some example items were as follows: “How often do you lose track of time while doing something you enjoy?” (Engagement, item 6), “To what extent do you receive help and support from others when you need it?” (Relationships, item 7), “To what extent do you feel that what you do in your life is valuable and worthwhile?” (Meaning, item 11), and “How often do you achieve the important goals you have set for yourself?” (Accomplishment, item 14). In Derakhshan and Datu’s (2025) study, Cronbach’s α for this scale was 0.93. In the current study, the SWS demonstrated good construct validity (x^2^/df = 3.511; CFI = 0.950; TLI = 0.932; SRMR = 0.051; RMSEA = 0.076) as well as excellent reliability (Cronbach’s α = 0.933).

### 3.6. Data Collection

Ethical approval for the present study was granted by the Ethics Committee of the School of Education, Xi’an International Studies University. Participants completed the self-report questionnaire in a single session. They were not subjected to any time restrictions. No form of compensation—whether financial or in the form of extra course credit—was provided. The students were informed that participation was entirely voluntary, and they could withdraw at any time without penalty or adverse consequences. Access to the questionnaire was contingent upon electronic informed consent, and respondents were guaranteed full anonymity and confidentiality of their data. Data were collected via Wenjuanxing, a widely used online survey platform in China.

### 3.7. Data Analysis

Before proceeding to the data analysis stage, a series of preliminary checks were conducted. A total of 520 questionnaires were collected. Missing values were first examined, outliers were identified, and the normality of the data was assessed. There were no missing values in the raw data. Prior to data analysis, all continuous variables were standardized to z-scores to screen for outliers. Following Hair et al. (2019), cases with absolute z-scores greater than 3 were identified as extreme outliers. These cases were then manually reviewed, and those found to contain careless, inconsistent, or illogical responses were excluded from subsequent analyses. Consequently, 440 questionnaires were retained for the final data analysis, resulting in a valid response rate of 84.6%. Then, skewness and kurtosis values were calculated to check the normality of the data. It was found that all skewness and kurtosis values fell between ±2, indicating that the data were in a normal distribution and suitable for further parametric analysis based on Kunnan (1998).

Next, the validity and reliability of the scales used in the current study were evaluated. The confirmatory factor analysis (CFA) was conducted to evaluate the validity of the scales. Model fit was assessed using several goodness-of-fit indices. A satisfactory model fit was indicated by χ^2^/df ratios between 1.0 and 5.0 (Woodrow, 2011), CFI and TLI values exceeding 0.90, and RMSEA and SRMR values below 0.08 (Bentler, 2007; L. T. Hu & Bentler, 1999). Cronbach’s α was used to evaluate the reliability of the scales. A coefficient above 0.70 indicated high reliability.

After that, correlations between teacher confirmation, L2 grit and well-being were calculated to confirm that the data met the prerequisites for mediation analysis. Subsequently, structural equation modeling (SEM) was performed using Amos 27.0 with 5000 bias-corrected bootstrap samples to examine whether L2 grit mediated the relationship between perceived teacher confirmation and student well-being. In the model, perceived teacher confirmation served as the independent variable, L2 grit as the mediator, and well-being as the dependent variable.

## 4. Results

### 4.1. Common Methods Bias

Harman’s one-way factor analysis was performed to test the common method bias. Six factors with eigenvalues greater than 1 were extracted, with the first factor explaining 37.23% of the total variance, well below the critical threshold of 40% (Podsakoff et al., 2003), suggesting that there was no common method bias in this study.

### 4.2. Descriptive Statistics and Correlation Analysis

As indicated in Table 1, all three constructs, including perceived teacher confirmation, L2 grit and well-being, were significantly and positively correlated with each other. The effect sizes were interpreted in terms of the thresholds proposed by Cohen (1988). Perceived teacher confirmation was positively correlated with L2 grit with a small effect size (r = 0.239; *p* < 0.01); L2 grit was positively correlated with well-being (r = 0.353; *p* < 0.01) with a medium effect size. Perceived teacher confirmation (r = 0.401; *p* < 0.01) was found to be positively associated with well-being, with a medium effect size. This suggested that higher levels of perceived teacher confirmation were associated with higher levels of L2 grit and a better sense of well-being.

### 4.3. The Mediation Analysis

Then, SEM was used to test the validity and significance of the potential mediating role of L2 grit in the interplay between perceived teacher confirmation and EFL learners’ sense of well-being. The SEM model demonstrated acceptable overall fit (x^2^/df = 2.835; CFI = 0.912; TLI = 0.906; SRMR = 0.0505; RMSEA = 0.065). A bootstrap sampling method using 5000 iterations with a 95% confidence interval of 95% was adopted. According to Table 2, the 95% confidence interval did not straddle zero, indicating the validity of the indirect path. Students’ perceptions of teacher confirmation positively and significantly predicted their sense of well-being (β = 0.250; *p* < 0.01). And teacher confirmation was a significant positive predictor of EFL learners’ L2 grit (β = 0.236; *p* < 0.01). L2 grit was a significant positive predictor of EFL learners’ well-being (β = 0.361; *p* < 0.001). L2 grit played a partial mediating role between perceived teacher confirmation and EFL learners’ sense of well-being (β = 0.059; *p* < 0.01). The multivariate correlation (R^2^ = 0.236) indicated that the model accounted for 23.6% of the variance.

## 5. Discussion

Based on the self-determination theory and the rhetorical/relational theory, the present study attempted to investigate the role of perceived teacher confirmation in promoting student well-being among Chinese EFL students. Furthermore, the potential mediating role of L2 grit between the two variables was examined. In this section, the results of this study are interpreted with reference to the relevant theoretical and empirical literature. The discussion is organized around each research question.

### 5.1. The Positive Role of Teacher Confirmation in EFL Learners’ Well-Being

The findings reveal that teacher confirmation is positively linked to EFL learners’ well-being, indicating that EFL learners who perceive higher levels of teacher confirmation tend to experience greater well-being. This finding is in accordance with prior research conducted on general education (e.g., LaBelle & Johnson, 2021). The finding further lends support to the rhetorical/relational goal theory (Mottet et al., 2006), which posits that positive interpersonal communication behaviors promote learners’ educational outcomes. This is possibly because when teachers create a confirming climate, students not only experience greater ease and comfort in their learning process but also exhibit reduced levels of negative emotions, such as apprehension and anxiety (Ellis, 2004; Hsu & Huang, 2017), thereby enhancing their sense of well-being. In addition, instructor-deployed confirming behaviors may contribute to students’ positive emotions, such as emotional interest, perceived emotional support, and favorable emotional valence toward the course (Goldman & Goodboy, 2014; Goodboy & Myers, 2008), which “broaden the scopes of attention and cognition, and, by consequence, initiate upward spirals toward increasing emotional well-being” (Fredrickson & Joiner, 2002, p. 172).

From the perspective of the self-determination theory, perceived teacher confirmation can satisfy learners’ three basic psychological needs, namely, autonomy, competence and relatedness (Ryan & Deci, 2020). Specifically, confirmatory instructional behaviors, encompassing the acknowledgment of student contributions, the provision of constructive feedback, and the validation of their learning efforts, are associated with an enhanced sense of autonomy in the learning process (Liu et al., 2024). Moreover, when students perceive higher levels of teacher confirmation, they are more confident about their abilities and potentialities to execute the tasks and activities in learning (Johnson & LaBelle, 2023; Peaslee, 2018), and their basic psychological need for competence will be fulfilled. Teachers’ employment of confirming behaviors can also create strong emotional bonds with their learners and provide them with a relaxed, enjoyable learning atmosphere (Burns et al., 2018; Rudick & Golsan, 2014), which may satisfy EFL learners’ need for relatedness. According to Ryan and Deci (2020), the satisfaction of learners’ basic psychological needs is conducive to students’ achievement, lifelong development and well-being.

### 5.2. The Mediating Role of L2 Grit in the Interplay Between Teacher Confirmation and Well-Being

The second objective of this study was to examine the potential mediating role of L2 grit in the relationship between perceived teacher confirmation and EFL learners’ well-being. The research findings indicate that L2 grit acts as a partial mediator. In other words, when EFL learners are confirmed by their language teachers as valuable and significant agents, they are empowered to persist in their academic endeavors and sustain interest in language learning regardless of difficulties and setbacks and, in turn, exhibit a greater sense of well-being. This finding is consistent with the broader literature showing that L2 grit mediates the association between teacher interpersonal behaviors (e.g., teacher care) and EFL learners’ sense of well-being (Zhou, 2023).

On the one hand, perceived teacher confirmation is found to be a positive and significant predictor of EFL learners’ L2 grit, which is in congruence with Bai and Zheng (2024). This suggests that when students perceive higher levels of acknowledgment and endorsement from their teachers, they will exhibit greater perseverance and more interest in learning a language. This finding provides empirical evidence to the theoretical assumptions that the employment of positive communication cues by language instructors in the classroom could cultivate learners’ positive character strengths (Mercer et al., 2018). Teachers’ confirming messages are conducive to strengthening the emotional bond between teachers and students as well as to the cultivation of an enjoyable learning environment (Houser & Hosek, 2018). According to Hejazi and Sadoughi (2023), when students are in an enjoyable learning environment, or experience enjoyment in foreign language learning, their grit will be fostered. This finding could also be explained by the Chinese educational context. Chinese teachers are considered authorities who provide more professional than emotional advice; teachers’ opinions are highly respected and rarely contradicted (Goldman et al., 2018). Thus, teachers’ confirming messages “act as stronger catalysts for persistent effort and continuous interest” (Bai & Zheng, 2024, p. 1973).

The results also indicate that L2 grit is a significant and positive predictor of student well-being, which is in line with previous studies conducted in the domain of language teaching and learning (e.g., He et al., 2023; Tang & Zhu, 2024; Tu & Shi, 2024; Yang, 2021). One possible explanation for this finding is that gritty individuals “demonstrate resilience and a strong commitment to achieving their goals, which can have a profound impact on their overall well-being” (Zhou, 2023). Moreover, as a positive personality trait, L2 grit is likely to yield a range of desirable educational outcomes for language learners, such as willingness to communicate, engagement and the improvement of language proficiency (e.g., Liu et al., 2024; Y. Wang & Derakhshan, 2023; Y. Wang & Kruk, 2024). These favorable educational outcomes make EFL learners have a greater sense of well-being. Hence, gritty EFL students experience happiness and well-being more than less gritty peers (Tu & Shi, 2024).

Finally, our study reveals a mediating role of L2 grit in the relationship between teacher confirmation and EFL learners’ sense of well-being. In terms of the rhetorical/relational goal theory, teachers’ interpersonal behaviors are conducive to fostering a positive teacher–student relationship and enhancing learners’ enjoyable learning experiences (Houser & Hosek, 2018), thus boosting EFL learners’ intrinsic motivation and self-efficacy beliefs about their abilities to learn a language well, as evidenced by previous studies (Goldman et al., 2018; Peaslee, 2018). Once they are highly motivated and have more confidence in L2 learning, they will be more ready to persevere even if they encounter difficulties and challenges in language learning (Fan & Cui, 2024; Fathi et al., 2024). Moreover, learners who are confirmed by their teachers as valuable individuals exhibit lower levels of apprehension (Ellis, 2004; Goldman et al., 2018) and higher levels of positive emotions (Goldman & Goodboy, 2014). These emotional states potentially contribute to the elevated levels of grit (Hejazi & Sadoughi, 2023), enabling them to buffer against foreign language learning burnout (X. Hu et al., 2025).

As a positive personality trait, grit can contribute to foreign language learning enjoyment (Khajavy & Aghaee, 2024; Pawlak et al., 2025). It can also promote learners’ persistence, self-regulation, and resilience against setbacks when confronting daily academic difficulties and challenges, thereby enhancing their level of learning engagement (Y. K. Wang, 2024). Furthermore, gritty L2 learners exhibit a higher level of mastery of language in various skills, such as linguistic proficiency (Demir, 2024; Teimouri, 2025), writing achievement (Fathi et al., 2024), vocabulary learning and extensive reading (Kramer et al., 2017). These favorable outcomes correspond respectively to the sub-dimensions of positive emotions, engagement, and accomplishment within the PERMA well-being framework.

## 6. Implications

The results of this inquiry have a number of theoretical and pedagogical implications. As far as the theoretical implications are concerned, the inquiry extends the existing literature on the interplay between teacher interpersonal behaviors and learner educational outcomes by highlighting the positive contribution of teachers’ confirming messages to EFL learners’ L2 grit and sense of well-being. To put it differently, the results of the present investigation further clarify the role of positive communication behaviors like confirmation. The findings also have important pedagogical implications for language teachers, teacher educators, educational administrators and policymakers. For language teachers, they should be aware of the significance of confirming behaviors in enhancing EFL learners’ perseverance and well-being in the challenging and demanding language-learning process. In language teaching, teachers may consciously employ confirming behaviors, such as taking time to listen to and answer students’ questions fully, showing interest in what students are learning, using an interactive teaching style, and providing oral or written feedback on students’ work, to create a confirming instructional climate. For teacher educators, they may develop learning resources, training programs or workshops to equip language teachers with specific skills and techniques to confirm their students. For school administrators and policy makers, teacher confirmation capabilities may be taken into account in their recruitment and evaluation of teachers.

## 7. Conclusions

Under the guidance of the self-determination theory and the rhetorical/relational goal theory, the current study investigated the role of teacher confirmation in predicting Chinese EFL learners’ well-being. Moreover, the mediating role of L2 grit between teacher confirmation and well-being was explored. Correlation analysis and SEM analysis indicate that perceived teacher confirmation is a positive predictor of EFL learners’ well-being, and L2 grit plays a partial mediating role in the interplay. Based on these findings, we may conclude that students’ grit and sense of well-being in learning English as a foreign language partially depend on teachers’ employment of interpersonal behaviors (e.g., confirming behaviors) in language-teaching environments. The findings of this study underscore the importance of teachers’ interpersonal cues in the cultivation of EFL learners’ personality traits and the increase in their sense of well-being.

## 8. Limitations and Directions for Future Research

The limitations of this study are as follows. First, while our hypothesized mediation model provides meaningful insights, we acknowledge that the cross-sectional design of this study precludes any causal interpretation among teacher confirmation, L2 grit, and well-being. The cross-sectional design also fails to capture the complexity and dynamics of well-being (Oxford, 2016). Longitudinal studies need to be designed in the future to address these issues. Second, relying solely on quantitative evidence, this study leaves room for follow-up qualitative work—via interviews, reflective journals or think-aloud protocols—to corroborate or interrogate the present results. In addition, the convenience sampling method used in the current study skewed toward female, English-major, and first-year students, limiting the generalizability of the findings. To strengthen future research, we advocate for replication studies that employ more rigorous sampling strategies to ensure a more balanced gender ratio, academic disciplines, and grade levels. Future studies may also investigate the contributions of other teacher interpersonal behaviors, such as classroom justice, teacher immediacy, closeness, and teacher clarity, to EFL learners’ well-being. Last, future studies may investigate the potential mediating roles of emotions (e.g., enjoyment, boredom, burnout, anxiety, hope, and pride) and personality traits (e.g., resilience and academic buoyancy) in the interplay between teacher confirmation and well-being.

## Figures and Tables

**Table 1:** Descriptive statistics and correlations between the variables (n = 440).

Variable	Mean	SD	Sk	Ku	TC	L2G	WB
**TC**	68.100	8.200	−0.088	−0.581	1		
**L2G**	27.516	4.778	0.357	0.027	0.239 **	1	
**WB**	56.880	7.767	0.264	0.160	0.401 **	0.353 **	1

Notes: ** *p* < 0.01; Sk refers to skewness; Ku refers to kurtosis; TC refers to teacher confirmation; L2G refers to L2 grit; WB refers to well-being.

**Table 2:** Direct and indirect effects of the variables.

Effect	Pathway	β	*p*	SE	95% Confidence Interval	
					Lower	Upper
Direct	TC → L2G	0.236	0.001	0.062	0.128	0.353
	L2G → WB	0.250	0.002	0.075	0.128	0.396
	TC → WB	0.361	0.000	0.055	0.259	0.466
Indirect	TC → L2G → WB	0.059	0.002	0.034	0.017	0.131
Total	TC → WB	0.420	0.000	0.044	0.329	0.500

Note: β refers to the standardized coefficient; TC refers to teacher confirmation; L2G refers to L2 grit; and WB refers to well-being.

## Data Availability

The data presented in this study are available on request from the corresponding author. The data are not publicly available due to privacy and ethical restrictions.
